# Efficient Esterification of Oxidized l-Glutathione and Other Small Peptides

**DOI:** 10.3390/molecules200610487

**Published:** 2015-06-08

**Authors:** Emily R. Vogel, William Jackson, Douglas S. Masterson

**Affiliations:** Department of Chemistry and Biochemistry, the University of Southern Mississippi, 118 College Drive #5043, Hattiesburg, MS 39406, USA; E-Mails: emily.vogel@eagles.usm.edu (E.R.V.); william.r.jackson@eagles.usm.edu (W.J.)

**Keywords:** esterification, peptide esterification, amino acids, carboxylic acids, esters

## Abstract

Oxidized l-glutathione was esterified to the tetra methyl ester using thionyl chloride in methanol solvent. Other alcohols were tested and the reaction progress was monitored via ESI-MS. This procedure proved to be compatible with other small peptides not containing serine and cysteine residues. In contrast to previously reported methods this procedure provided convenient access to esterified peptides requiring no purification, extended reaction times, or complicated reaction setups.

## 1. Introduction

The design and synthesis of novel glutathione analogues are studied extensively for their pharmacological properties in the treatment of a wide range of diseases [1,2,3]. Reduced glutathione (GSH), γ-l-glutamyl-l-cysteinyl-glycine, is prone to reactivity at the sulfhydryl, terminal amino group, and both carbonyls. Thus, the synthesis of GSH analogues are a synthetic challenge to chemists, and the clever manipulation of protecting groups are needed to prevent unwanted reactions. One approach to synthesize protected GSH analogues is to start with the readily available disulfide dimmer of GSH, l-glutathione (GSSG) **1**. GSSG is affordable and the sulfhydryl functional groups are protected in their disulfide form. **1** can be inconveniently converted to **2a** by Fischer esterification [4,5,6] resulting in the protection of the carboxyl groups. In our hands the reported Fischer esterification protocol required extensive purification and the isolated yield was too low to be of practical value. Although **2a** is commercially available the cost is quite high ($1290 per 100 mg) making direct purchase prohibitive in many cases [7]. Additional esterification strategies include utilizing trimethylsilyl chloride in methanol solvent [2,8] and the use of diazomethane to form methyl esters [9,10]. However, the use of diazomethane is toxic, potentially explosive, and requires specialized glassware not readily available in all labs. 

**Scheme 1:**
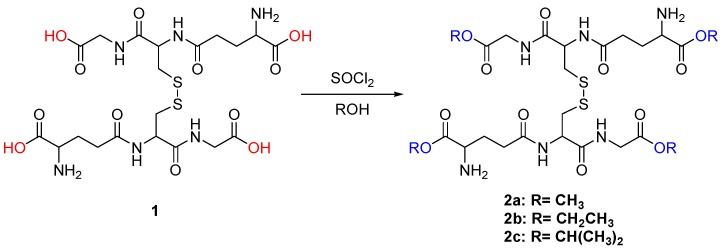
Preparation of oxidized glutathione tetra-alkyl esters (GSSG(OR)_4_) from oxidized glutathione (GSSG) using thionyl chloride in alcohol solvent.

Herein, we report a convenient synthesis of **2a** using thionyl chloride in methanol solvent as illustrated in Scheme 1. Surprisingly, this method eliminated side reactions, long reaction times, and column purification. Furthermore, we extended the method to other peptides in order to gain an understanding of the compatibility with other protogenic amino acids. The ease with which peptide esters are formed could be an invaluable tool for peptide mass spectrometry [3,11,12,13] as the protected carboxylic acid enhances signal intensity.

## 2. Results and Discussion

The esterification of oxidized glutathione (GSSG) with thionyl chloride was previously reported [14], but studies monitoring the reaction conversion have not been reported. The disposition toward thionyl chloride acylation is likely due to the incompatibilities often associated with the harsh conditions of thionyl chloride which are subject to both side reactions and incomplete formation of product. Therefore, we set out to study the limitations of this method by monitoring the reaction using ESI-MS in the presence of various alcohols using **1** as a substrate. 

**Figure 1:**
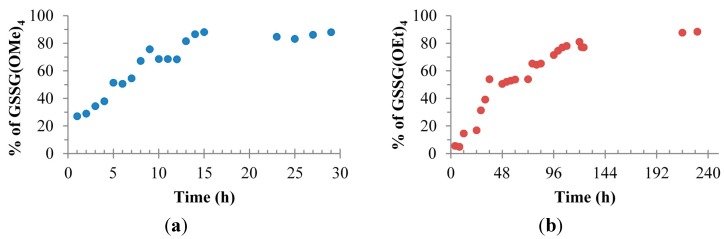
(**a**) Plot of the percent conversion to the GSSG tetra esters *vs.* time in methanol as determined by ESI-MS (**b**) Plot of the percent conversion to the GSSG tetra esters *vs.* time in ethanol as determined by ESI-MS.

The reactions were performed on 250 mg of GSSG in 50 mL of anhydrous alcohol. To this solution 2.5 mL of thionyl chloride was added slowly and placed into a refrigerator at 4 °C. At various time intervals a 1.0 mL aliquot was taken, concentrated under reduced pressure, suspended into 1% acetic acid solution (50% methanol/water), and analyzed via ESI-MS. The relative intensities of the *m/z* for the tetra-ester were plotted as a percentage of ester products as illustrated in Figure 1. The procedure worked well in methanol solvent reaching completion within 16 h. The same experimental conditions were applied using an excess of anhydrous ethanol resulting in complete conversion to **2b** in 144 h. In contrast, when the same experimental conditions were applied using an excess of anhydrous isopropyl alcohol only a 14% conversion to **2c** was realized along with significant formation of various unidentified side products. We therefore concluded that studying other 2° or even 3° alcohols would likely not be productive.

To further demonstrate the utility of this method the reaction conditions were applied to other small peptides as shown in Table 1. These peptides were chosen based on comparable size to GSSG and the coverage of amino acids present in their sequences. The reactions were performed on a 1.0 µmol scale and immediately concentrated following a 24 h incubation at 4 °C. The peptides were then immediately analyzed via ESI-MS. 

**Table 1:** Peptides subjected to the thionyl chloride esterification with methanol and analyzed via ESI-MS.

Peptide ^a^	Peptide Sequence	Product Entry	Complete Conversion	Estimated % ^c^ Conversion
l-glutathione oxidized	2QCG ^b^	**2**	**Yes**	**100% ^d^**
l-glutathione reduced	QCG	**3**	**No**	**26%**
Fibronectin Analog	GRADSPK	**4**	**No**	**23%**
Bradykinin (1–7)	RPPGFSP	**5**	**No**	**63%**
Necrofibrin, rat	WTVPTA	**6**	**No**	**64%**
[d-Ala2,d-Met5]-Enkephalin	YAGFM	**7**	**No**	**90%**
Angiotensin II, human	DRVYIHPF	**8**	**Yes**	**100%**
Thymopentin (TP-5)	RKDVY	**9**	**Yes**	**100%**
Neurotensin (9–13)	RPYIL	**10**	**Yes**	**100%**
[Ile3]-Pressinoic acid	CYIQNC ^b^	**11**	**No**	**94%**

^a^ Reaction conditions of the peptides were run using 3.44 M thionyl chloride in methanol at 4 °C for 24 h; ^b^ Peptide contains a disulfide bridge between the two cysteine residues; ^c^ Yields estimated by taking the sum or relative intensities of all product peaks over the total relative intensities observed in ESI-MS in excess of 5% relative abundance; ^d^ isolated product yield.

The reactions listed in Table 1 proceeded smoothly under the esterification protocol in most cases. However, the peptides containing serine, fibronectin and bradykinin, appear to have been converted to the esterified alkyl chloride derivative at the serine residue [15,16,17]. This is evidenced by the *m/z* corresponding to the molecular formula of the esterified peptide with chlorine replacing the hydroxyl functional group and the signature isotopic ratio of chlorine. These results are not unexpected considering that thionyl chloride is used to transform primary alcohols into alkyl chlorides. However, we did observe the esterified product containing the unaltered serine residue as a minor constituent in the product mixture. Interestingly, the threonine containing peptide, necrofibrin, underwent smooth conversion to the ester without converting the 2° alcohol into an alkyl chloride. The phenol functional group of tyrosine in [DAla2,DMet5]-Enkephalin, Angiotensin II, Thymopentin (TP-5), and Neurotensin (9–13) did not interfere with the esterification procedure.

The free sulfhydryl of the cysteine residue also proved to be problematic when subjected to the esterification protocol. Reduced l-glutathione was subjected to the reaction conditions producing the desired esterified peptide as a minor constituent. The reaction mixture contained significant quantities of **2** and several other unidentified species by ESI-MS analysis. However, the methionine residue of Enkephalin and the disulfide bond of [Ile3]-Pressinoic acid and GSSG were cleanly converted to their respective esters. In addition, the residues of aspartic acid and glutamic acid were converted to their respective methyl esters as expected. Despite these limitations all of the other protogenic amino acids withstood the reaction conditions and were easily isolated and characterized without the need for extensive purification. 

## 3. Experimental Section 

### 3.1. General Experimental 

NMR spectra were acquired on a Bruker 400 MHz NMR in proton decoupled mode. The internal standard used in the NMR experiment was the residual solvent signal for CD_3_OD. ESI MS was carried out on a ThermoFisher LXQ ESI-Ion trap mass spectrometer using Optima LCMS grade methanol and water from Fisher Scientific. The methanol used for the esterification reactions was distilled from calcium hydride, absolute ethanol was stored over molecular sieves, and 2-propanol distilled from sodium prior to use. l-oxidized glutathione and reduced glutathione were obtained from Sigma-Aldrich. All other peptides were acquired from the American Peptide Company and used as received. Peptides furnished with Certificate of Analysis (COA) contained trace impurities and those masses were provided courtesy of American Peptide Company. Mass spectra and NMR spectra can be found in the supplementary materials section.

### 3.2. Synthesis of GSSG and GSH Peptides in Alcohol Solutions 

#### 3.2.1. Synthesis of Compound **2**

In a 125 mL Erlenmeyer flask, 0.250 g of l-oxidized glutathione (0.408 mmol) was added to 50 mL of freshly distilled methanol and capped with a rubber septum. The contents were placed into an ice bath and allowed to cool to 0 °C. The addition of 2.5 mL of thionyl chloride (34.4 mmol) was by syringe, the flask was swirled, and placed into a refrigerator at 4 °C. After 24 h the reaction was concentrated under reduced pressure resulting in 0.272 g of product (100% yield).The product was analyzed via ESI MS. ESI MS of [M + H]^+^ calculated [C_24_H_44_N_6_O_12_S_2_]^+^, 669.22, found 669.2. ^1^H-NMR (400MHz, CD_3_OD) δ = 4.67 (m, 2H), 4.05 (m, 2H), 3.88 (s, 4H), 3.76 (s, 6H), 3.62 (s, 6H), 3.21 (s, 4H), 2.93–2.81 (m, 2H), 2.49 (m, 4H), 2.12 (m, 4H) and ^13^C-NMR (100MHz, CD_3_OD) δ = 174.3, 173.0, 171.6, 170.7, 54.0, 53.9, 53.7, 52.8, 42.0, 41.4, 32.3, 27.0. Spectra are consistent with published data [18].

#### 3.2.2. Synthesis of Compound **2b**

In a 125 mL Erlenmeyer flask, 0.250 g of l-oxidized glutathione (0.408 mmol) was added to 50 mL of absolute ethanol and capped with a rubber septum. The contents were placed into an ice bath and allowed to cool to 0 °C. The addition of 2.5 mL of thionyl chloride (34.4 mmol) was by syringe, the flask was swirled, and placed into a refrigerator at 4 °C. After 24 h the reaction was concentrated under reduced pressure resulting in 0.295 g of product (100% yield). The solid was taken and characterized by ESI-MS. ESI-MS of [M + H]^+^ calculated [C_28_H_44_N_6_O_12_S_2_]^+^, 725.28, found 725.25. ^1^H-NMR (400 MHz, CD_3_OD) δ = (400 MHz, MeOD) δ 4.78 (dd, *J* = 9.6, 4.6 Hz, 2H), 4.33 (q, *J* = 7.1 Hz, 4H), 4.19 (q, *J* = 7.1 Hz, 4H),4.13(m, 2H), 3.97 (d, *J* = 2.2 Hz, 4H), 3.28 (dd, *J* = 14.0, 4.6 Hz, 2H), 2.99 (dd, *J* = 13.9, 9.7 Hz, 2H), 2.61 (t, *J* = 7.1 Hz, 4H), 2.23 (m, *J* = 21.7, 14.6, 7.5 Hz, 4H), 1.35 (t, *J* = 7.1 Hz, 6H), 1.28 (t, *J* = 7.2 Hz, 6H).^13^C-NMR (100MHz, CD_3_OD) δ =174.4, 173.0, 171.1, 170.2, 63.4,62.4, 53.9,53.7, 42.1, 41.3, 32.6, 27.0, 14.5, 14.43. Spectra are consistent with published data [19].

#### 3.2.3. Synthesis of Compound **2c**

In a 125 mL Erlenmeyer flask, 0.250 g of l-oxidized glutathione (0.408 mmol) was added to 50 mL of freshly distilled 2-propanol and capped with a rubber septum. The contents were placed onto an ice bath and allowed to cool to 0 °C. The addition of 2.5 mL of thionyl chloride (34.4 mmol) was by syringe, and the flask was swirled and placed into a refrigerator at 4 °C. After 24 h the reaction was concentrated under reduced pressure. The resulting solid was taken and characterized by ESI-MS. ESI-MS of [M + H]^+^ calculated [C_32_H_57_N_6_O_12_S_2_]^+^, 781.35, found 781.25. Expected mass not present in significant quantities so no NMR data was recorded. 

#### 3.2.4. Synthesis of Compound **3**

In a 125 mL Erlenmeyer flask, 0.250 g of l-reduced glutathione (0.813 mmol) was added to 50 mL of freshly distilled anhydrous methanol and capped with a rubber septum. The contents were placed onto an ice bath and allowed to cool to 0 °C. The addition of 2.5 mL of thionyl chloride (34.4 mmol) was by syringe, and the flask was swirled and placed into a refrigerator at 4 °C. After 24 h the reaction was concentrated under reduced pressure. The resulting solid was taken and characterized by ESI-MS. ESI-MS of [M + H]^+^ calculated [C_12_H_22_N_3_O_6_S]^+^, 336.12, found 336.08. Sample contained multiple impurities so no NMR analysis was performed.

### 3.3. General Synthesis of Peptides in Methanol Solutions 

The conditions for each peptide were scaled down from the GSSG reaction study in methanol based on the number of carboxylic acid groups present in the molecule. The amount of methanol used in the reaction was 756 equivalents of methanol to which 105 equivalents of thionyl chloride was added for each carboxylic acid present in the peptide. This results in a 3.44 M solution of thionyl chloride in methanol. All reactions were performed on 1.0 mg of peptide and incubated at 4 °C for 24 h. At the end of the incubation the crude material was concentrated and analyzed by ESI-MS.

#### 3.3.1. Synthesis of Compound **4**

In a dry glass vial fitted with a cap 1.0 mg of Fibronectin (1 µmol) was dissolved into 83.9 µL of anhydrous methanol (2.075 mmol) and placed in an ice bath at 0 °C. To the solution 21.0 µL of thionyl chloride (0.289 mmol) was added slowly via syringe. The vial was shaken and placed into a refrigerator at 4 °C. After 24 h the sample was concentrated under reduced pressure, dissolved into 1.0 mL of 1% acetic acid in a 1:1 solution of methanol:water, and analyzed on the ESI-MS. ESI-MS of [M + H]^+^ calculated [C_31_H_56_N_11_O_11_]^+^, 758.42, found 758.42.

#### 3.3.2. Synthesis of Compound **5**

In a dry glass vial fitted with a cap 1.0 mg of Bradykinin (1 µmol) was dissolved into 40.5 µL of anhydrous methanol and placed in an ice bath at 0 °C. To the solution 10.1 µL of thionyl chloride (0.14 mmol) was added slowly via syringe. The vial was shaken and placed into the fridge at 4 °C. After 24 h the sample was concentrated under reduced pressure, dissolved into 1.0 mL of 1% acetic acid in a 1:1 solution of methanol:water, and analyzed on the ESI-MS. ESI-MS of [M + H]^+^ calculated [C_36_H_55_N_10_O_9_]^+^, 771.41, found 771.38. 

#### 3.3.3. Synthesis of Compound **6**

In a dry glass vial fitted with a cap 1.0 mg of necrofibrin, rat (1 µmol) was dissolved into 45.5 µL of anhydrous methanol (1.12 mmol) and placed in an ice bath at 0 °C. To the solution 11.4 µL of thionyl chloride (0.156 mmol) was added slowly via syringe. The vial was shaken and placed into a refrigerator at 4 °C. After 24 h the sample was concentrated under reduced pressure, dissolved into 1.0 mL of 1% acetic acid in a 1:1 solution of methanol:water, and analyzed on the ESI-MS. ESI-MS of [M + H]^+^ calculated [C_33_H_50_N_7_O_9_]^+^, 688.37; found 688.20.

#### 3.3.4. Synthesis of Compound **7**

In a dry glass vial fitted with a cap 1.0 mg of [DAla2,DMet5] Enkephalin (2 µmol) was dissolved into 52.1 µL of anhydrous methanol (1.29 mmol) and placed in an ice bath at 0 °C. To the solution 13.0 µL of thionyl chloride (0.18 mmol) was slowly added via syringe. The vial was shaken and placed into a refrigerator at 4 °C. After 24 h the sample was concentrated under reduced pressure, dissolved into 1.0 mL of 1% acetic acid in a 1:1 solution of methanol:water, and analyzed on the ESI-MS. ESI-MS of [M + H]^+^ calculated [C_29_H_40_N_5_O_7_S]^+^, 602.26, found 602.1. 

#### 3.3.5. Synthesis of Compound **8**

In a dry glass vial fitted with a cap 1.0 mg of angiotensin II, human (1 µmol) was dissolved into 58.6 µL of anhydrous methanol (1.45 mmol) and placed in an ice bath at 0 °C. To the solution 14.6 µL of thionyl chloride (0.20 mmol) was added slowly via syringe. The vial was shaken and placed into a refrigerator at 4 °C. After 24 h the sample was concentrated under reduced pressure, dissolved into 1.0 mL of 1% acetic acid in a 1:1 solution of methanol:water, and analyzed on the ESI-MS. ESI-MS of [M + H]^+^ calculated [C_52_H_76_N_13_O_12_]^+^, 1074.57, found 1074.6. 

#### 3.3.6. Synthesis of Compound **9**

In a dry glass vial fitted with a cap 1.0 mg of Thymopentin (TP-5) (1 µmol) was dissolved into 90.1 µL of anhydrous methanol (2.22 mmol) and placed in an ice bath at 0 °C. To the solution 22.5 µL of thionyl chloride (0.289 mmol) was added slowly via syringe. The vial was shaken and placed into the fridge at 4 °C. After 24 h the sample was concentrated under reduced pressure, dissolved into 1.0 mL of 1% acetic acid in a 1:1 solution of methanol:water, and analyzed on the ESI-MS. ESI-MS of [M + H]^+^ calculated [C_32_H_54_N_9_O_9_]^+^, 708.40, found 708.47. Masses present in starting material from COA: 341(14%), 680 (100%), 1358(8%).

#### 3.3.7. Synthesis of Compound **10**

In a dry glass vial fitted with a cap 1.0 mg of Thymopentin (TP-5) (1 µmol) was dissolved into 90.1 µL of anhydrous methanol (2.22 mmol) and placed in an ice bath at 0 °C. To the solution 22.5 µL of thionyl chloride (0.289 mmol) was added slowly via syringe. The vial was shaken and placed into the fridge at 4 °C. After 24 h the sample was concentrated under reduced pressure, dissolved into 1.0 mL of 1% acetic acid in a 1:1 solution of methanol:water, and analyzed on the ESI-MS. ESI-MS of [M + H]^+^ calculated [C_32_H_54_N_9_O_9_]^+^, 708.40, found 708.47. 

#### 3.3.8. Synthesis of Compound **11**

In a dry glass vial fitted with a cap 1.0 mg of [Ile3] Pressinoic Acid (1 µmol) was dissolved into 41.3 µL of anhydrous methanol (1.02 mmol) and placed in an ice bath at 0 °C. To the solution 10.3 µL of thionyl chloride (0.14 mmol) was added slowly via syringe. The vial was shaken and placed into a refrigerator at 4 °C. After 24 h the sample was concentrated under reduced pressure, dissolved into 1.0 mL of 1% acetic acid in a 1:1 solution of methanol:water, and analyzed on the ESI-MS. ESI-MS of [M + H]^+^ calculated [C_31_H_47_N_8_O_10_S_2_ ]^+^,755.29, found 755.22. 

## 4. Conclusions 

We have prepared GSSG methyl esters in high yield requiring no purification or complex reaction protocols. The title compound could also be prepared as the ethyl ester at the expense of increased reaction time. Based on these findings the reaction conditions were applied to other small peptides and found to be highly compatible with many peptide sequences. We believe this procedure will find significant use in the area of peptide modification for both synthetic and analytical purposes.

## Supplementary Materials

Supplementary materials can be accessed at: http://www.mdpi.com/1420-3049/20/06/10487/s1.
