# Supplementary material for: Efficient Esterification of Oxidized l-Glutathione and Other Small Peptides
Source: Molecules. 2015 Jun 8;20(6):10487–95. doi: 10.3390/molecules200610487 (PMC6272753; doi:10.3390/molecules200610487)
Supplement: Supplementary file 1 [file molecules-20-10487-s001.pdf]

Supplemental Information for:

An Efficient Esterification of Oxidized L-Glutathione and Other Small Peptides

General Experimental Conditions

NMR spectra were acquired on a Bruker 400 MHz NMR in proton decoupled mode. The internal standard used in the NMR experiment was residual solvent signal for CD_3OD. ESI MS was carried out on a ThermoFisher LXQ ESI-Ion trap mass spectrometer using Optima LCMS grade methanol and water from Fisher Scientific. All samples were analyzed using a 1% acetic acid 1:1 methanol:water solution. The methanol used for the esterification reactions was distilled from calcium hydride, absolute ethanol was stored over molecular sieves, and 2-propanol distilled from sodium prior to use. L-oxidized glutathione and reduced glutathione was obtained from Sigma-Aldrich, and all other peptides were acquired from the American Peptide Company and used as received. Peptides furnished with Certificate of Analysis (COA) contained trace impurities and those masses were provided courtesy of American Peptide Company.

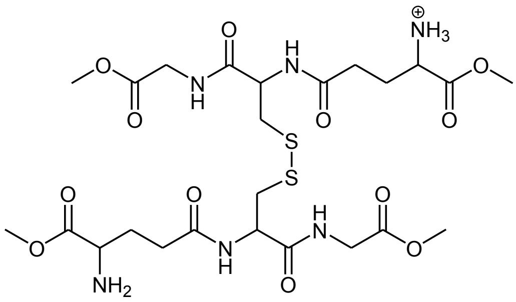

Glycine, L-γ-glutamyl-l-cysteinyl-1,3-dimethyl ester, bimol. (2→2′)-disulfide (2) ESI MS of $[M + H]^+$ calculated $[C_{24}H_{44}N_6O_{12}S_2]^+$, 669.22, found 669.2. ^1H-NMR and ^{13}C-NMR matched published data [1].

Observed Mass (*m/z*)	Relative Abundance (%)	Comments	Number of Esters
669.25	100.00%	$[M + H]^+$	4
655.25	24.50%	$[M + H]^+$	3
335.17	18.32%	$[M + 2H]^{2+}$	4
328.17	7.56%	$[M + 2H]^{2+}$	3

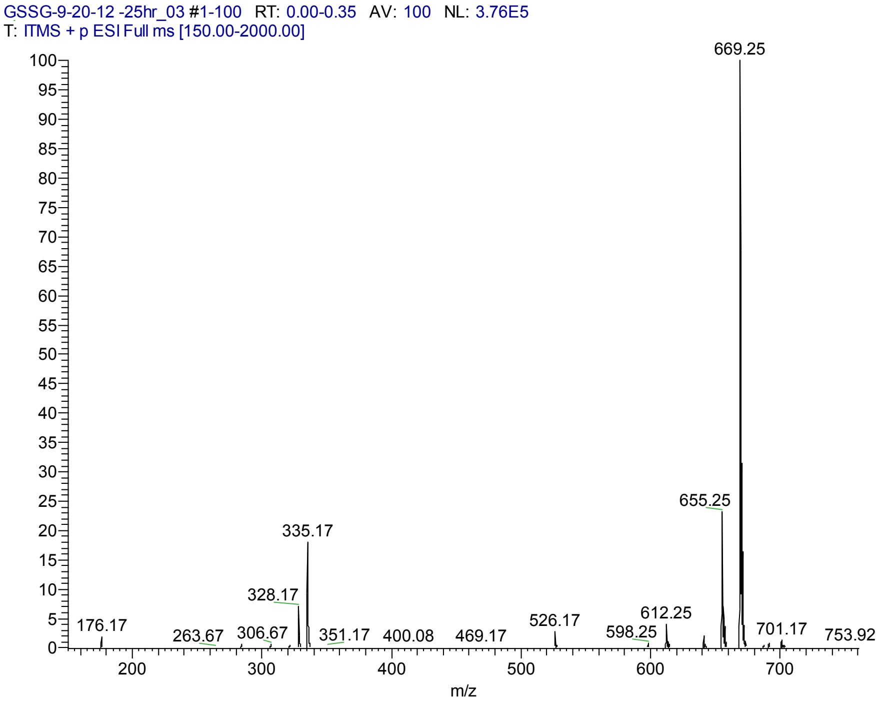

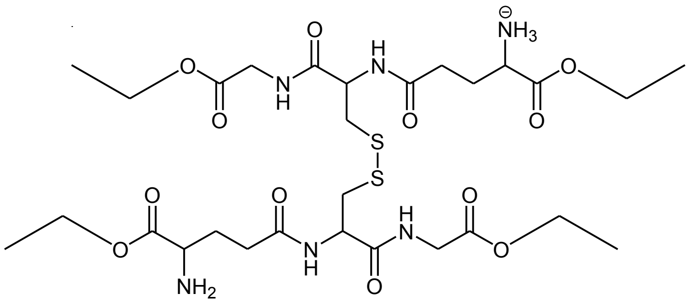

Glycine, L-γ-glutamyl-L-cysteinyl-1,3-diethyl ester, bimol. (2→2′)- ESI MS of $[M + H]^+$ calculated $[C_{28}H_{44}N_6O_{12}S_2]^+$, 725.28, found 725.25. ^{1}H-NMR and ^{13}C-NMR matched published data [2].

Observed Mass (*m/z*)	Relative Abundance (%)	Comments	Number of Esters
725.25	100%	$[M + H]^+$	4
363.17	24.00%	$[M + 2H]^{2+}$	4
697.25	7.05%	$[M + H]^+$	3

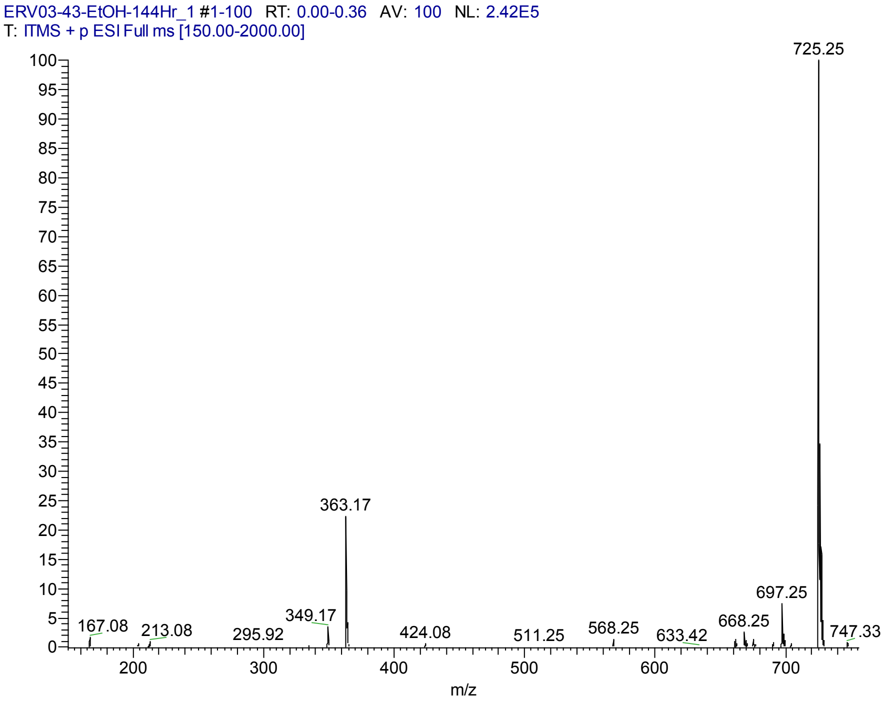

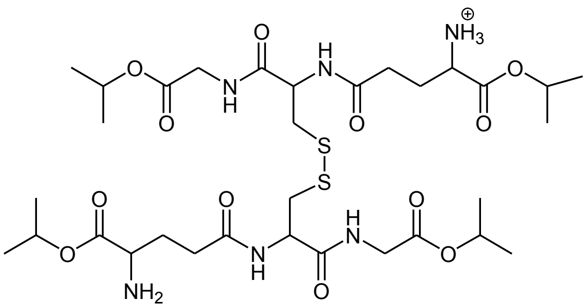

Glycine, L-γ-glutamyl-L-cysteinyl-1,3-diisopropyl ester, bimol. (2→2′)- ESI MS of $[M + H]^+$ calculated $[C_{32}H_{57}N_6O_{12}S_2]^+$, 781.35, found 781.25.Expected mass not present in significant quantities so no NMR data was recorded.

Observed Mass (*m/z*)	Relative Abundance (%)	Comments	Number of Esters
697.25	100.00%	$[M + H]^+$	2
349.17	78.04%	$[M + 2H]^{2+}$	2
739.25	74.30%	$[M + H]^+$	3
370.17	62.30%	$[M + 2H]^{2+}$	3
208.83	27.00%	UK	UK
781.25	9.00%	$[M + H]^+$	4
391.17	7.50%	$[M + H]^{2+}$	4

UK = unknown.

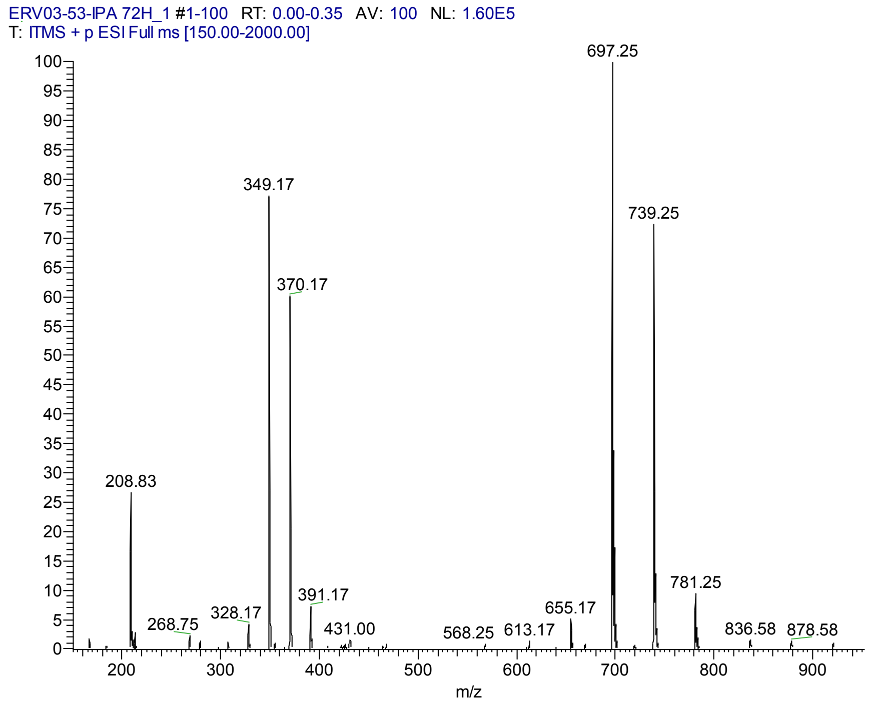

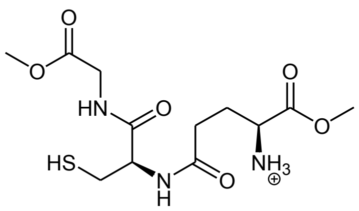

ESI MS of $[M + H]^+$ calculated $[C_{12}H_{22}N_3O_6S]^+$, 336.12, found 336.08. Sample contained multiple impurities so no NMR analysis was performed.

**Observed Mass (*m/z*)**	**Relative Abundance (%)**	**Comments**	**Number of Esters**
318.17	100%	UK	UK
336.08	56.70%	$[M + H]^+$	2
669.17	26.40%	$[*M + H]^+$	4
701.17	13.25%	UK	UK
304.17	8.30%	UK	UK
655.164	5.80%	$[*M + H]^+$	3
261.17	5.50%	UK	UK

*M = $[C_{24}H_{44}N_6O_{12}S_2]^+$, Oxidized Glutathione; UK = unknown.

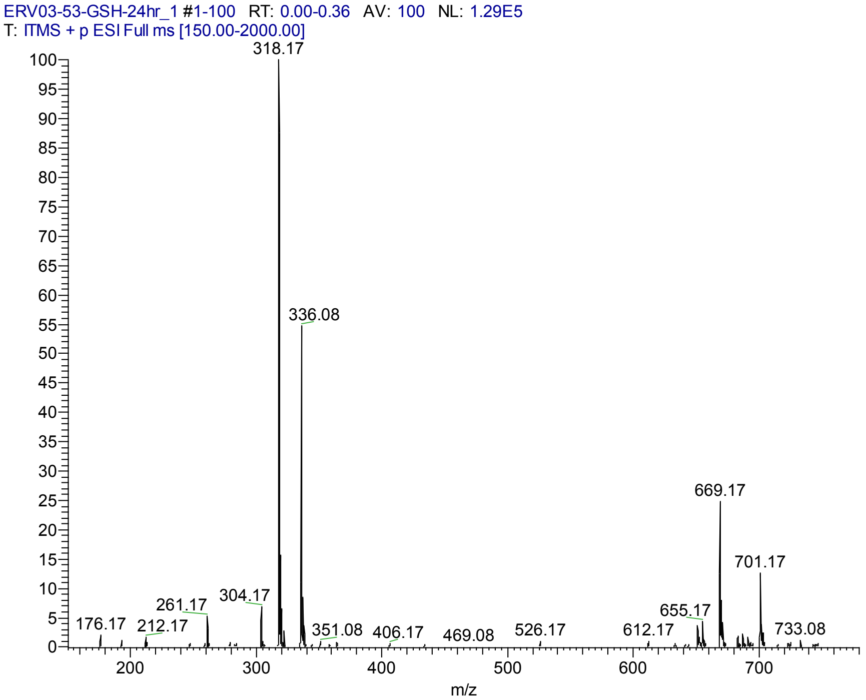

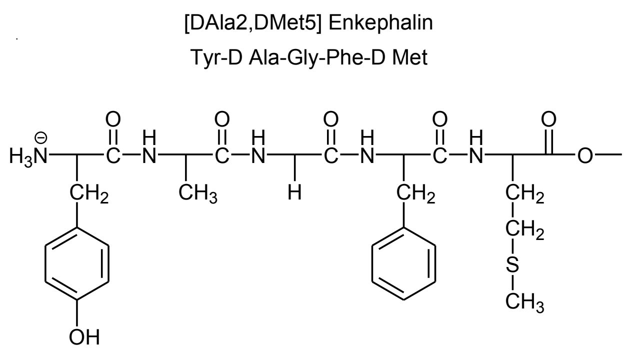

ESI MS of $[M + H]^+$ calculated $[C_{29}H_{40}N_5O_7S]^+$, 602.26, found 602.1. Masses present before reaction: 411 (8%), 587 (100%), 610 (23%), 1174 (42%).

Observed Mass (*m/z*)	Relative Abundance (%)	Comments
602.17	100%	$[M + H]^+$
1202.67	54.00%	$[2M + H]^+$
616.08	16.54%	UK

UK = unknown.

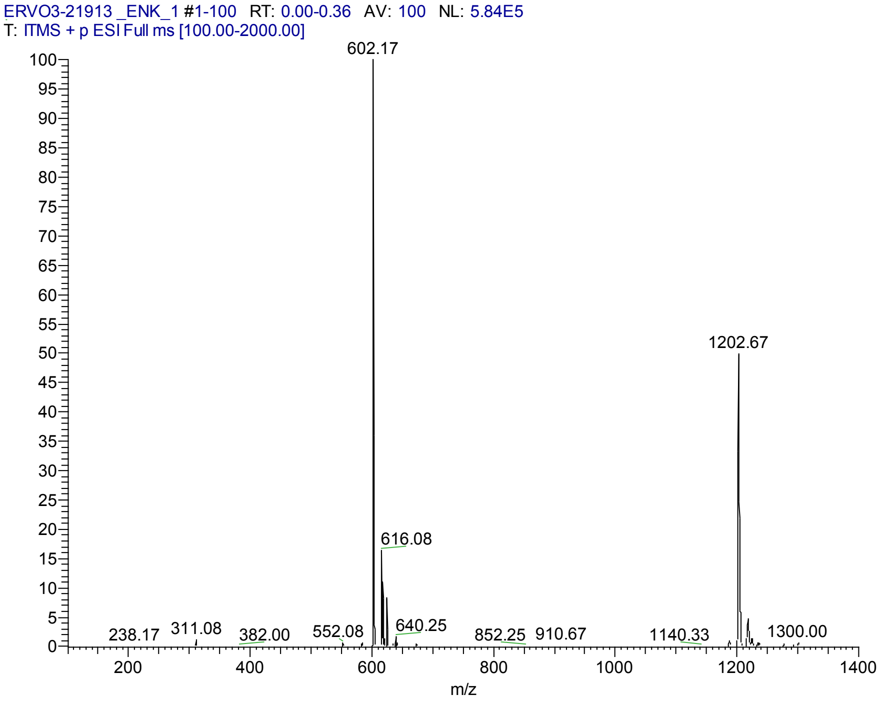

[Ile3]-Pressinoic Acid

Cys-Tyr-Ile-Gln-Asn-Cys

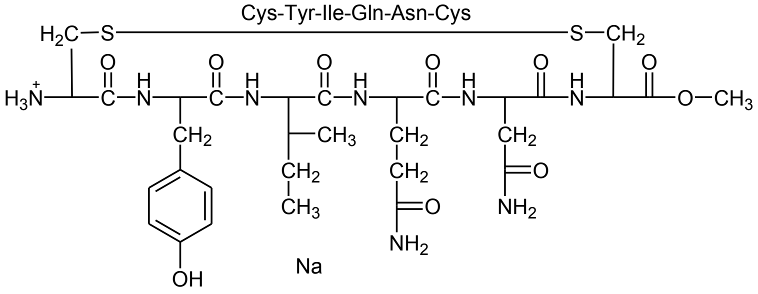

ESI MS of $[M + H]^+$ calculated $[C_{31}H_{47}N_8O_{10}S_2]^+$, 755.29, found 755.22. Masses present before reaction: 726 (10%), 742 (100%), 764 (21%), 781 (16%), 1483 (10%), 1522 (20%).

**Observed Mass (*m/z*)**	**Relative Abundance (%)**	**Comments**
755.25	100.00%	$[M + H]^+$
777.25	50.00%	$[M+ Na]^+$
1509.08	24.55%	$[2M+ H]^+$
770.212	10.54%	UK

UK = unknown.

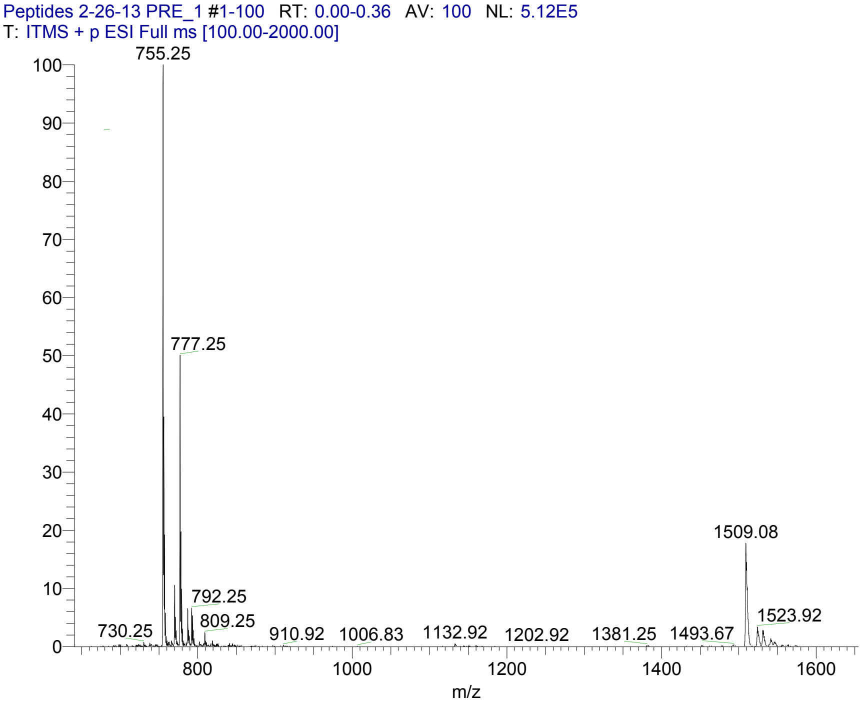

Angiotensin II, human
Asp-Arg-Val-Tyr-Ile-His-Pro-Phe

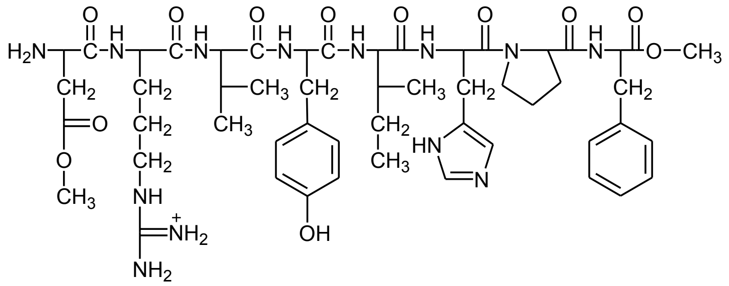

ESI MS of $[M + H]^+$ calculated $[C_{52}H_{76}N_{13}O_{12}]^+$, 1074.57, found 1074.6 *m/z*.

**Observed Mass (*m/z*)**	**Relative Abundance (%)**	**Comments**
1074.58	100.00%	$[M + H]^+$
537.83	35.34%	$[M+ 2H]^{2+}$

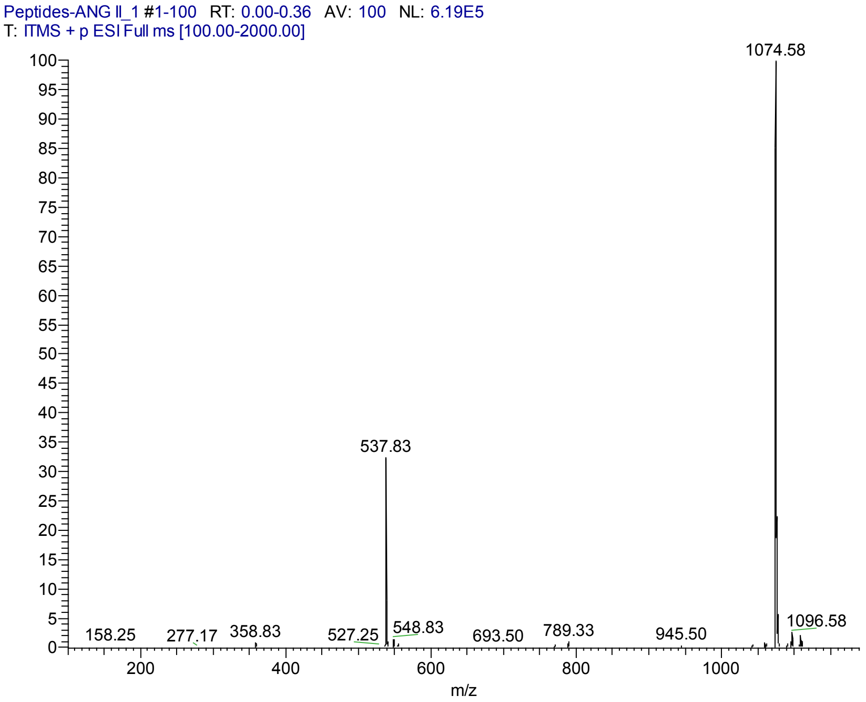

Fibronectin

Gly-Arg-Ala-Asp-Ser-Pro-Lys

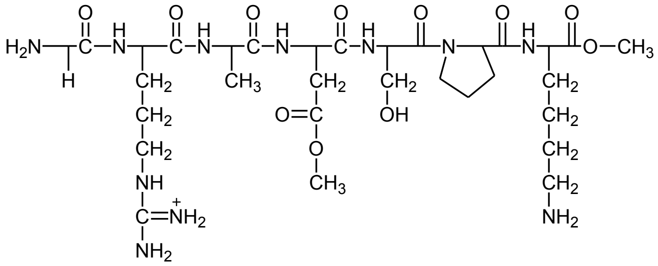

ESI MS of $[M + H]^+$ Calculated $[C_{31}H_{56}N_{11}O_{11}]^+$, 758.42, Found 758.42.

Observed Mass (*m/z*)	Relative Abundance (%)	Comments
388.75	100.00%	$[{*M} + 2H]^{2+}$
776.42	85.40%	$[{*M} + H]^+$, ^{35}Cl
758.42	39.66%	$[M + H]^+$
778.365	33.97%	$[{*M} + H]^+$, ^{37}Cl
379.75	25.28%	$[M + 2H]^{2+}$
345.25	22.36	UK
446.25	20.94	UK
537.83	9.43%	UK

*M = $C_{31}H_{55}ClN_{11}O_{10}$; UK = unknown.

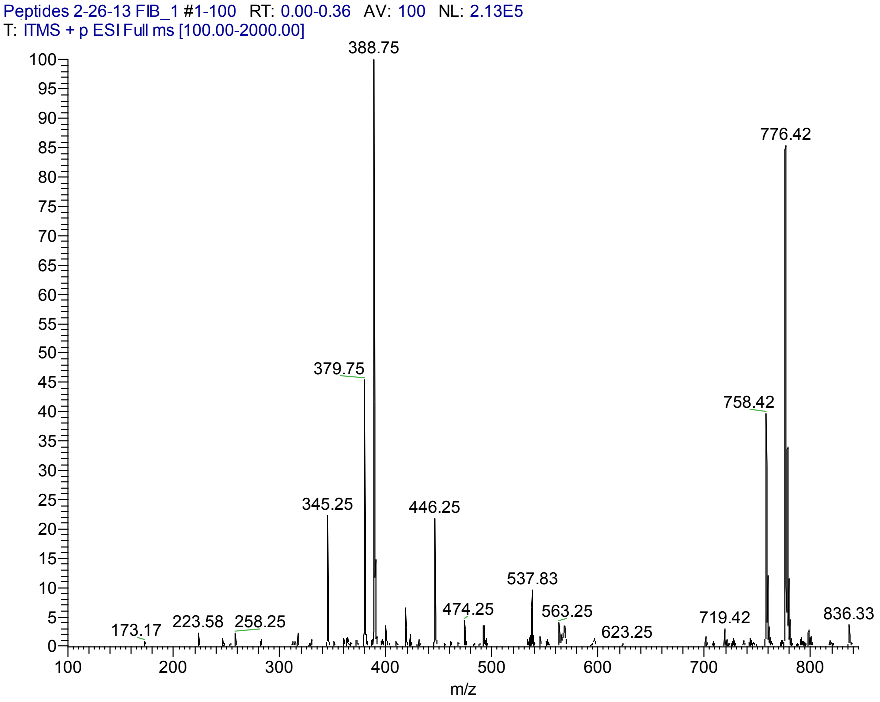

Necrofibrin, rat
Trp-Thr-Val-Pro-Thr-Ala

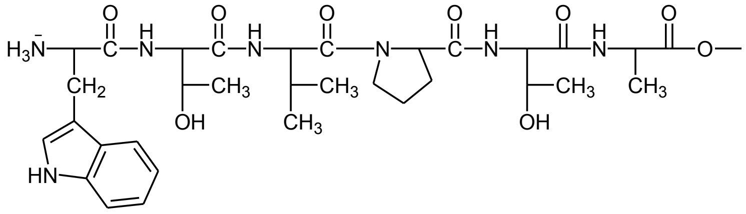

ESI MS of $[M + H]^+$ calculated $[C_{33}H_{50}N_7O_9]^+$, 688.37; found 688.20. Masses present in starting material: 674 (55%), 696 (100%), 1346 (19%), 1368 (10%).

Observed Mass (*m/z*)	Relative Abundance (%)	Comments
688.25	100.00%	$[M + H]^+$
* 710.33	37.07%	$[*M+ H]^+$
335.678	18.46%	$[*M+ 2H]^{2+}$
344.51	16.51%	$[M+ 2H]^{2+}$
1374.58	9.4%	$[**M+ H]^+$

* Impurity from starting material undergoing methyl esterification *m/z* = 696; ** Impurity from starting material undergoing two methyl esterifications *m/z* = 1346.

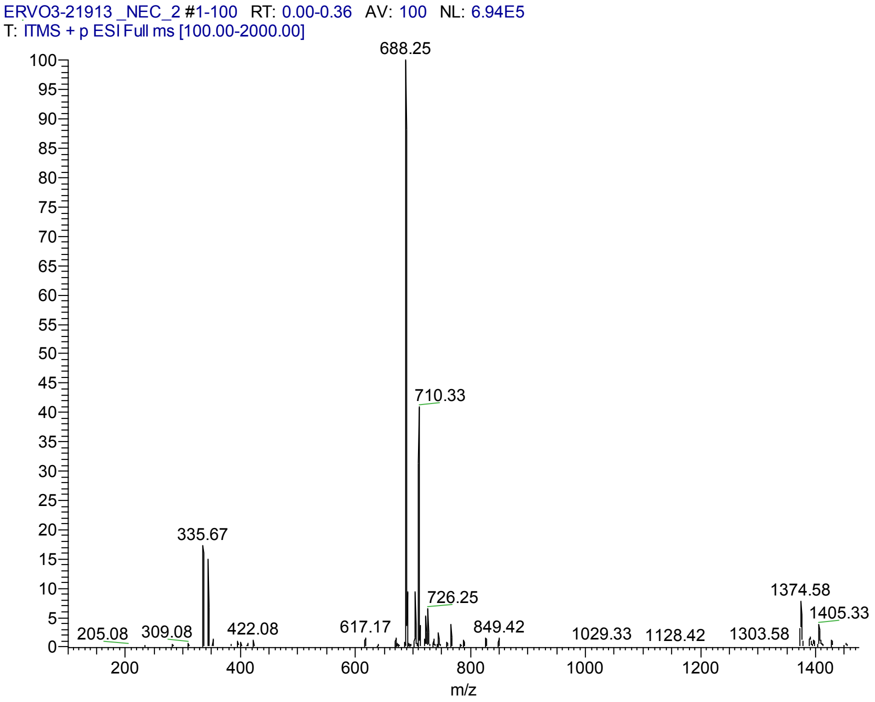

Neurotensin (9-13)
Arg-Pro-Tyr-Ile-Leu

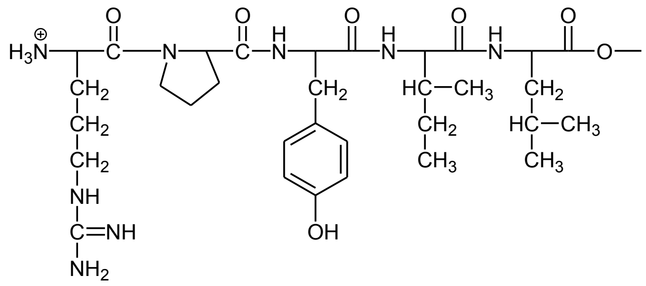

ESI MS of $[M + H]^+$ calculated $[C_{33}H_{55}N_8O_7]^+$, 675.42; found 675.39. Masses present in starting material: 661 (100%), 1320 (56%), 1322 (40%).

Observed Mass (*m/z*)	Relative Abundance (%)	Comments
675.42	100.00%	$[M + H]^+$

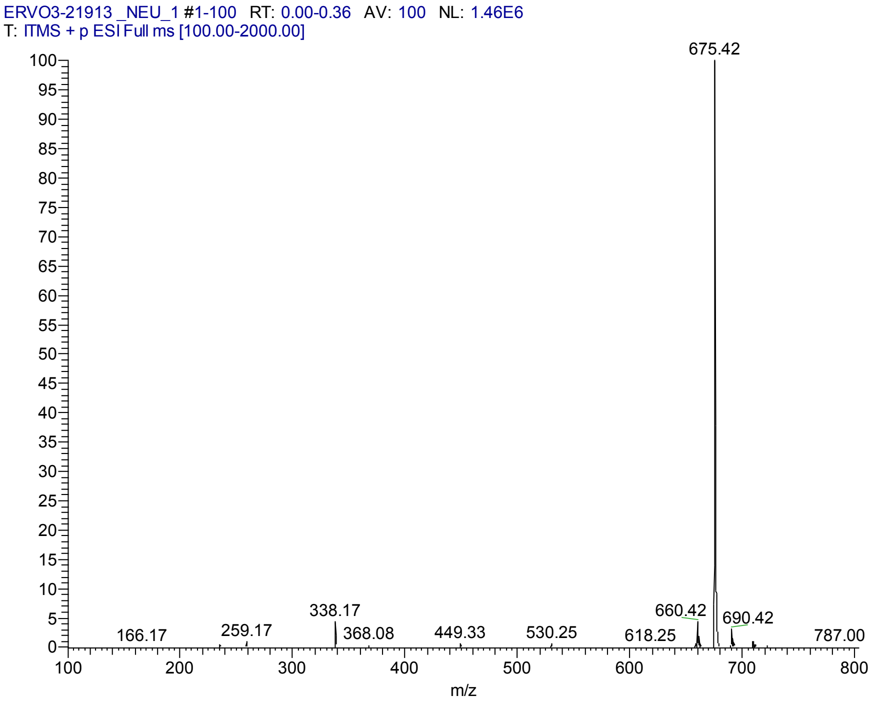

Thymopentin (TP-5)
Arg-Lys-Asp-Val-Tyr

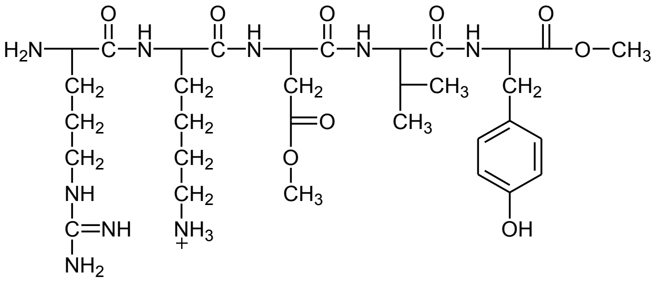

ESI MS of $[M + H]^+$ calculated $[C_{32}H_{54}N_9O_9]^+$, 708.40, found 708.47. Masses present in starting material: 341(14%), 680 (100%), 1358(8%).

Observed Mass (*m/z*)	Relative Abundance (%)	Comment
708.42	100.00%	$[M + H]^+$
354.76	25.40%	$[M+ 2H]^{2+}$

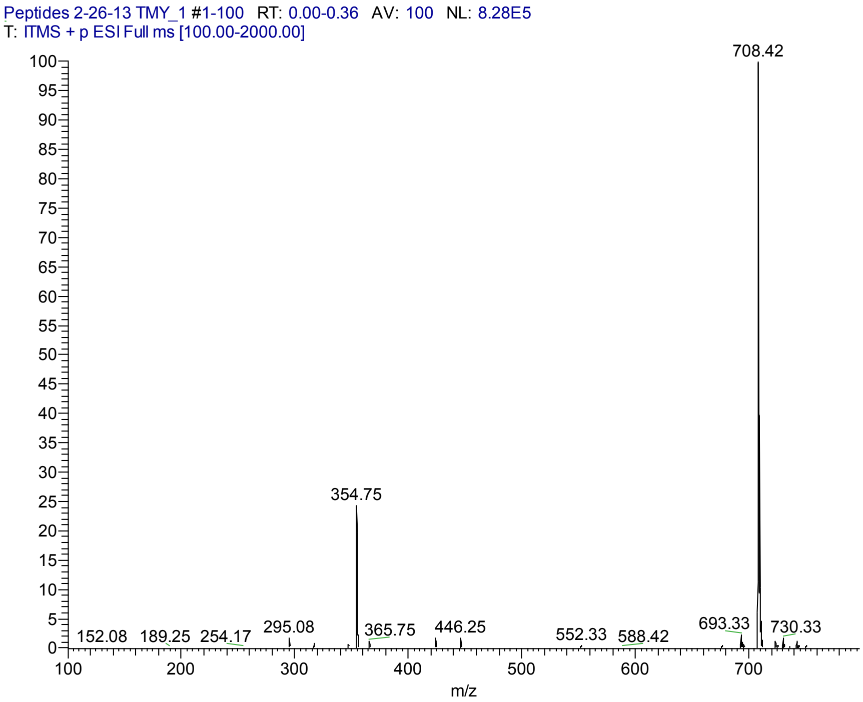

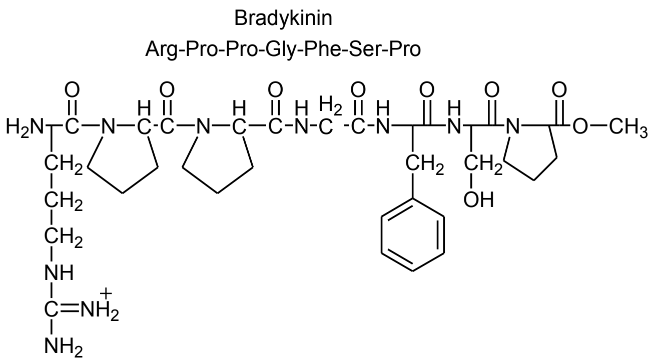

ESI MS of $[M + H]^+$ calculated $[C_{36}H_{55}N_{10}O_9]^+$, 771.41, found 771.38.

**Observed Mass (*m/z*)**	**Relative Abundance (%)**	**Comment**
771.42	100.00%	$[M + H]^+$
789.33*	49.97%	$[*M + H]^+$, ^{35}Cl
791.34*	21.34%	$[*M + H]^+$, ^{37}Cl
587.33	7.26%	UK

*M = $C_{36}H_{54}ClN_{10}O_8$; UK = unknown.

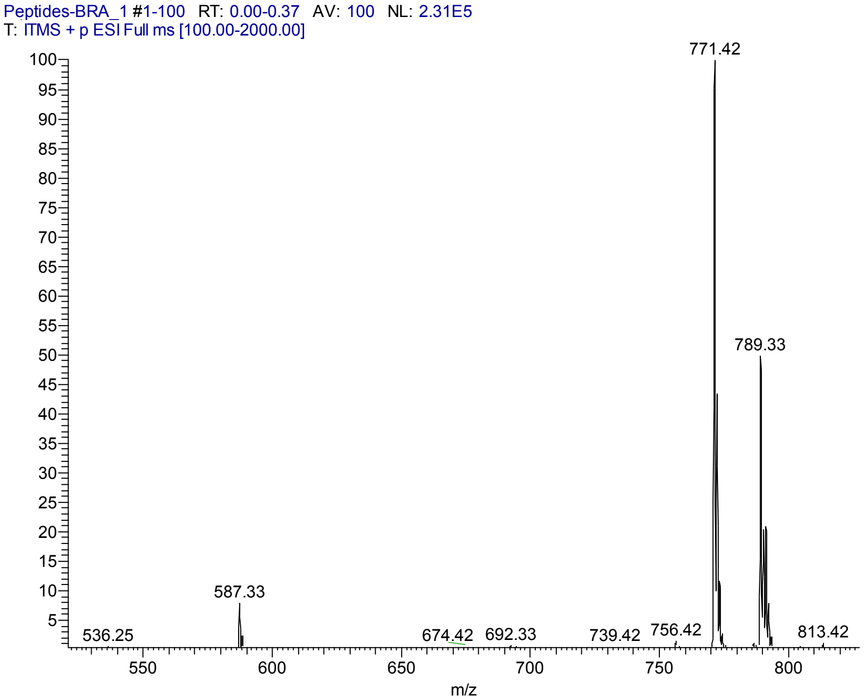

NMR DATA

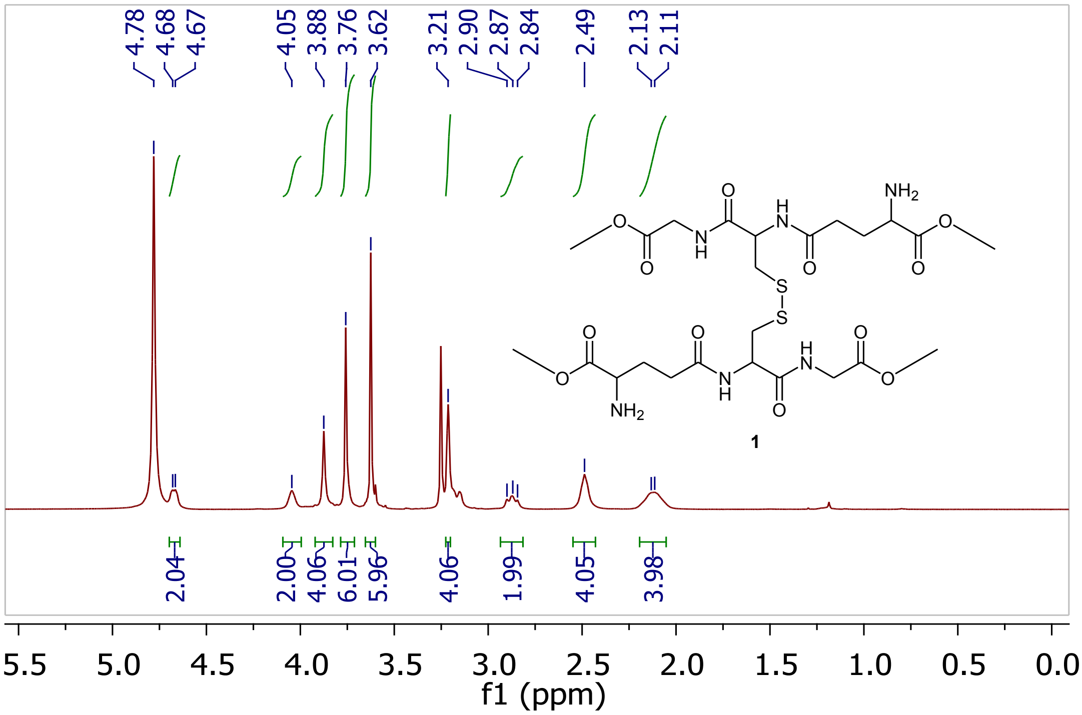

4.78
4.68
4.67
4.05
3.88
3.76
3.62
3.21
2.90
2.87
2.84
2.49
2.13
2.11
2.04
2.00
4.06
6.01
5.96
4.06
1.99
4.05
3.98
NH2
O
N
H
S
1
5.5 5.0 4.5 4.0 3.5 3.0 2.5 2.0 1.5 1.0 0.5 0.0
f1 (ppm)

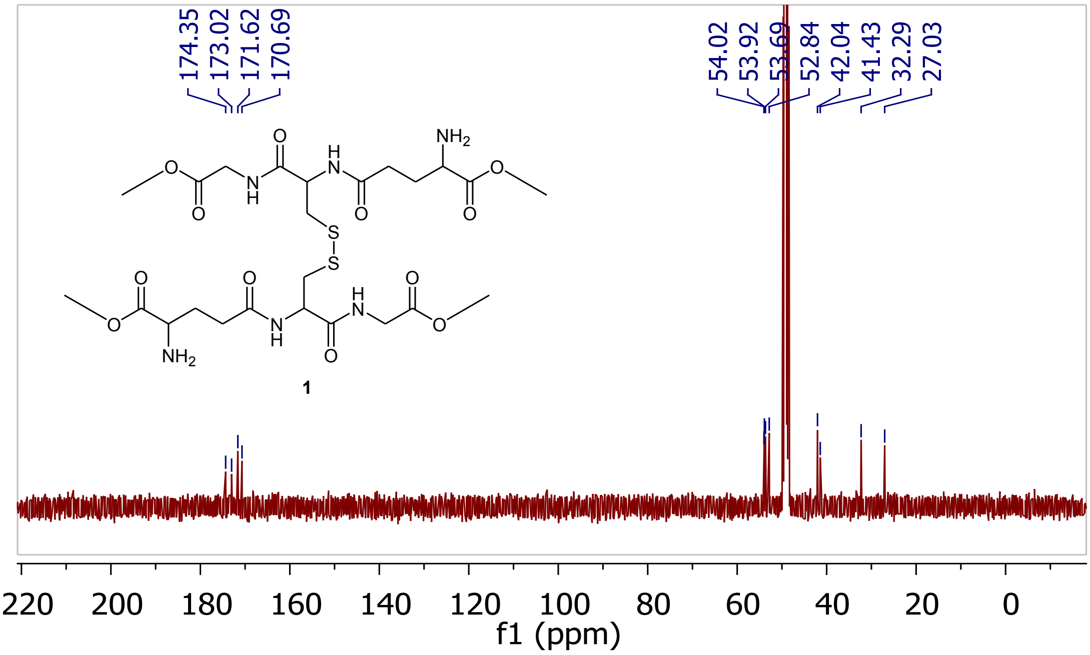

174.35
173.02
171.62
170.69
54.02
53.92
53.69
52.84
42.04
41.43
32.29
27.03
NH2
O
N
H
S
1
220 200 180 160 140 120 100 80 60 40 20 0
f1 (ppm)

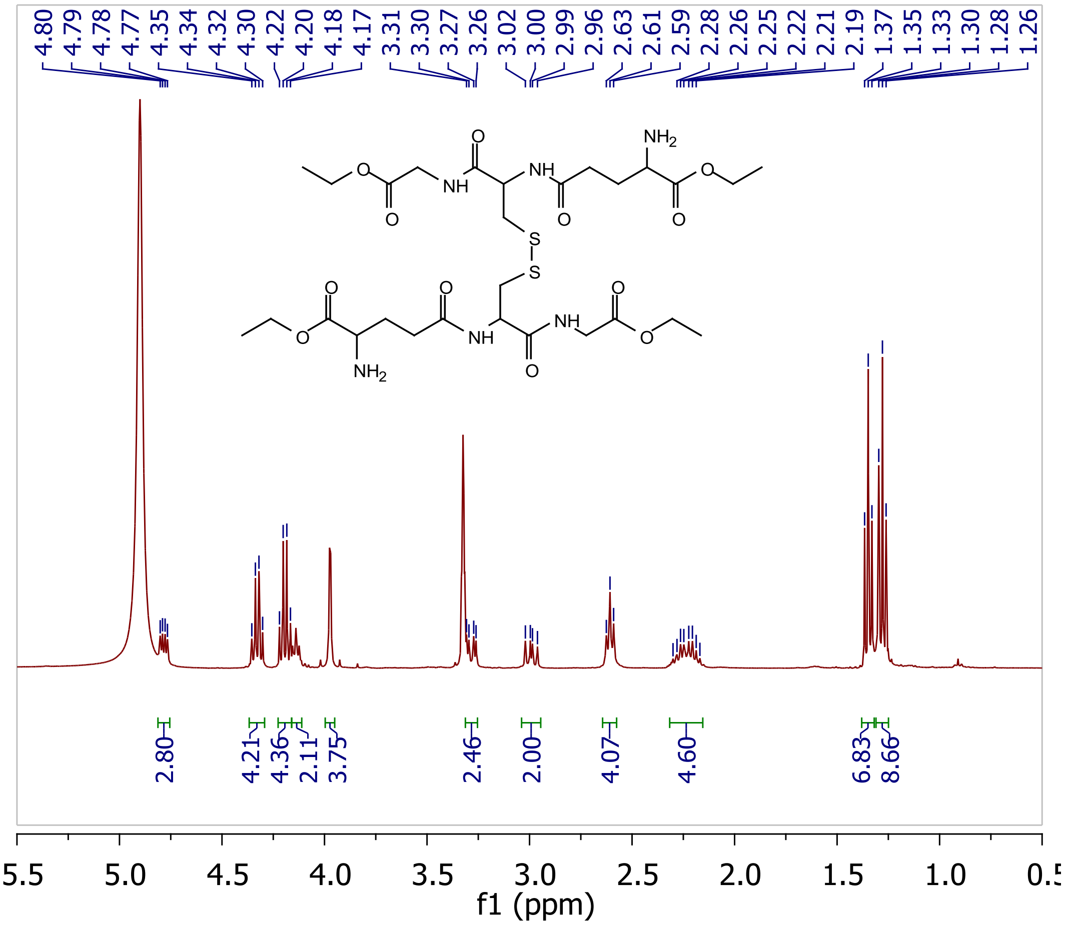
4.80 4.79 4.78 4.77 4.35 4.34 4.32 4.30 4.22 4.20 4.18 4.17 3.31 3.30 3.27 3.26 3.02 3.00 2.99 2.96 2.63 2.61 2.59 2.28 2.26 2.25 2.22 2.21 2.19 1.37 1.35 1.33 1.30 1.28 1.26
NH2
O
NH
O
S
S
NH
2.80 4.21 4.36 2.11 3.75 2.46 2.00 4.07 4.60 6.83 8.66
f1 (ppm)

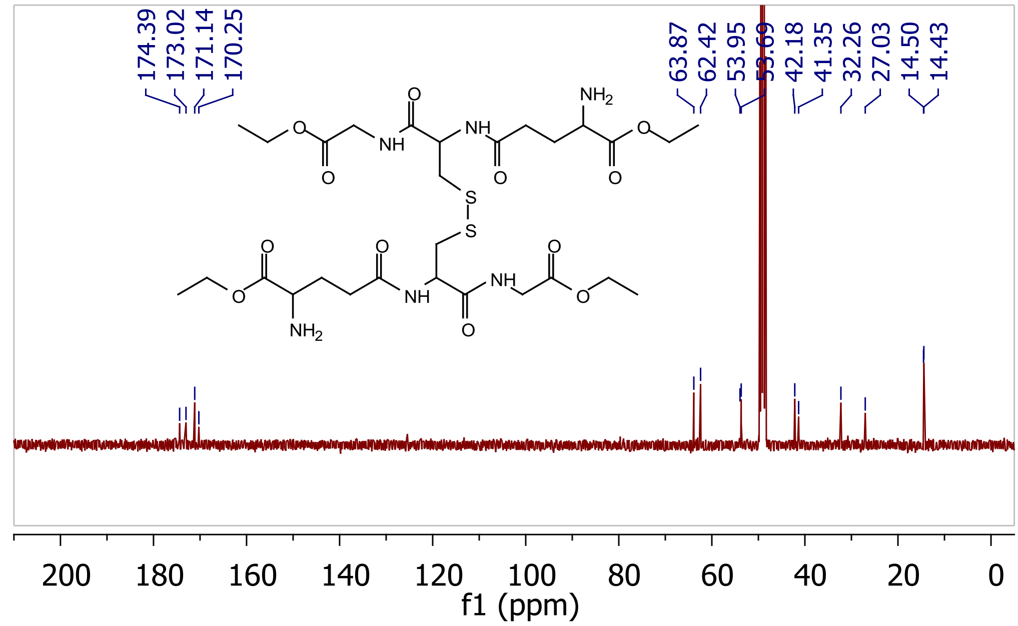
174.39 173.02 171.14 170.25
63.87 62.42 53.95 53.69 42.18 41.35 32.26 27.03 14.50 14.43
NH2
O
NH
S
f1 (ppm)

Reference

1. Gatterdam, V.; Ramadass, R.; Stoess, T.; Fichte, M.A.H.; Wachtveitl, J.; Heckel, A.; Tampé, R. Three-Dimensional Protein Networks Assembled by Two-Photon Activation. *Angew. Chem. Int. Ed.* **2014**, *53*, 5680–5684.
2. McCulloch, M.W.B.; Coombs, G.S.; Banerjee, N.; Bugni, T.S.; Cannon, K.M.; Harper, M.K.; Veltri, C.A.; Virshup, D.M.; Ireland, C.M. Psammaplin A as a general activator of cell-based signaling assays via HDAC inhibition and studies on some bromotyrosine derivatives. *Bioorg. Med. Chem.* **2009**, *17*, 2189–2198.
